# A genomic predictor for age at sexual maturity for mammalian species

**DOI:** 10.1111/eva.13635

**Published:** 2024-01-10

**Authors:** Matthew J. Heydenrych, Alyssa M. Budd, Benjamin Mayne, Simon Jarman

**Affiliations:** ^1^ School of Biological Sciences University of Western Australia Crawley Western Australia Australia; ^2^ Environomics Future Science Platform, Indian Ocean Marine Research Centre Commonwealth Scientific and Industrial Research Organisation (CSIRO) Crawley Western Australia Australia; ^3^ School of Molecular and Life Sciences Curtin University Bentley Western Australia Australia

**Keywords:** CpG density, elastic net regression, epigenetics, machine learning, mammals, sexual maturity

## Abstract

Age at sexual maturity is a key life history trait that can be used to predict population growth rates and develop life history models. In many wild animal species, the age at sexual maturity is not accurately quantified. This results in a reduced ability to accurately model demography of wild populations. Recent studies have indicated the potential for CpG density within gene promoters to be predictive of other life history traits, specifically maximum lifespan. Here, we have developed a machine learning model using gene promoter CpG density to predict the mean age at sexual maturity in mammalian species. In total, 91 genomes were used to identify 101 unique gene promoters predictive of age at sexual maturity across males and females. We found these gene promoters to be most predictive of age at sexual maturity in females (*R* ^2^ = 0.881) compared to males (*R* ^2^ = 0.758). The median absolute error rate was also found to be lower in females (0.427 years) compared to males (0.785 years). This model provides a novel method for species‐level age at sexual maturity prediction without the need for long‐term monitoring. This study also highlights a potential epigenetic mechanism for the onset of sexual maturity, indicating the possibility of using epigenetic biomarkers for this important life history trait.

## 1 INTRODUCTION

Sexual maturity is an important life history trait for wildlife management (Tacha et al., 1989). The age at first reproduction or average age at sexual maturity (ASM) can be used to estimate a range of population outcomes, including the growth potential and extinction risk of populations (Grandi et al., 2010; Kramer et al., 1999; Luba et al., 2020; Onyango et al., 2013). Both ASM and the age at first reproduction, combined with the age at reproductive senescence or death can provide estimates for the reproductive potential of cohorts and, therefore, estimates for entire populations (Kemper et al., 2014; Mackey et al., 2006). This information can be used by conservation and resource managers to determine minimal viable populations sizes, to manage fishery catch limits and to determine the potential for population recovery (Hobday & Ryan, 1997; Luba et al., 2020; Wang et al., 2019).

There are a range of methods to determine sexual maturity. These commonly involve biopsy sampling of an animal's gonads followed by reproductive cell assessment (Falahatkar et al., 2013; Luetjens & Weinbauer, 2012; Mecklenburg et al., 2019; Vidal et al., 2021). Once individual assessments of sexual maturity have been obtained, the sample ages can be used to obtain a population or species‐level ASM estimate. Long‐term sampling approaches have also been attempted to estimate ASM, using mother‐daughter pairing and kinship assessments (Levasseur et al., 2021). Molecular processes, such as hormone level shifts, are also known to be involved in both sexual differentiation and maturity and could provide potentially useful biomarkers (Navarro‐Martín et al., 2009). A recent study in wild mice assessed the steroid hormones corticosterone, testosterone and progesterone in mouse hair and compared hormone levels to sexual maturity, though a test for sexual maturity was not developed (Carlitz et al., 2019). Similarly, links between testosterone and gonadotropin and sexual maturity were explored in lampreys and in captive dugongs, but application to a wild animal was not attempted (Burgess et al., 2013; Larsen, 1987). Additionally, studies have been carried out identifying ASM in cetaceans from hormone profiles of blood and from faecal material (Kjeld et al., 1992, 2003; Rolland et al., 2005). Many of these methods are invasive, require post‐mortem study or necessitate intense sampling efforts that are not feasible for large‐scale species‐level assessments (Kramer et al., 1999). Identifying alternative rapid and minimally invasive methods for species ASM determination is, therefore, an important goal for ongoing wildlife management.

Molecular based methods for the prediction of important life history traits have been used in a broad variety of species. For example, sex can be determined in various species across vertebrate groups, including birds and mammals, using basic PCR methods where sex‐specific genomic differences occur (Ennis & Gallagher, 1994; Jensen et al., 2003). The age of an animal can be predicted by measuring DNA methylation, the addition of a methyl group to cytosine‐phosphate‐guanosine (CpG) sites (De Paoli‐Iseppi et al., 2017; Tanabe et al., 2020). The CpG density in gene promoters has a role in the regulation of gene expression but has also been shown to be both associated and predictive of lifespan (Budd et al., 2023; Mayne et al., 2019; McLain & Faulk, 2018; Tamagawa et al., 2017). A recent study has used DNA methylation analysis to estimate various life history traits, including maximum lifespan, gestational time and age at sexual maturity (Lu et al., 2023). These studies highlight that DNA methylation patterns, specifically CpG density in promoter regions of genes, are correlated with life history traits such as lifespan and indicate the potential for similar approaches for estimating ASM.

In this study, we develop a genomic‐based predictor for ASM in mammals. We calculate the CpG density of gene promoters from 91 mammalian genomes with linked age at sexual maturity information. This work further expands on the utilization of epigenetic and genomic‐based methods for the prediction of life history traits. To our knowledge, this is the first study to develop a genomic‐based method to predict the age at sexual maturity in mammals.

## 2 METHODS

### 2.1 Species with sexual maturity information and reference genomes

Species‐level mean sexual maturity age data were obtained from AnAge: The Animal Ageing and Longevity Database at the Human Aging and Genomic Resources repository (Tacutu et al., 2018). This dataset provided sexual maturity age information for both sexes as well as maximum lifespan estimates and was accessed on the 23rd of August 2022. The AnAge age and sexual maturity information has been used in various publications and is provided as a curated and quantitative dataset, where various filtering and data quality checks are adhered to and averaging across species has been performed where necessary (De Magalhães & Costa, 2009). A list of mammalian chromosome level genome assemblies was retrieved from the National Library of Medicine's National Centre for Bioinformatics (NCBI) database (https://www.ncbi.nlm.nih.gov/genome/browse#!/eukaryotes/) on the 23rd of August 2022 (O'Leary et al., 2016). The genome browse tool was employed with filters for ‘Animal’ as the group, ‘Mammal’ as the subgroup and ‘Chromosome’ as the assembly level filter. Chromosome level assemblies were used to prevent the use of incomplete and non‐contiguous genome assemblies without the need to perform additional computational analyses. Using chromosome level assemblies allowed us to rapidly determine if CpG density is predictive of age at sexual maturity in mammals. This list of available chromosomal genomes was matched against the AnAge dataset. Species with both genomes and sexual maturity information were included in the study. Four subsets of the data were created to generate predictive models. These comprised species with female and male age at sexual maturity information (male and female subsets; SupplementaryInfo_9_pd_Female; SupplementaryInfo_11_pd_Male), and species with lifespan estimates available in AnAge were also used to generate a ratio of sexual maturity to lifespan for each sex (male and female ratio subsets; SupplementaryInfo_10_pd_FemaleRatio; SupplementaryInfo_12_pd_MaleRatio). Species where no maximum lifespan was recorded were dropped from the ratio subsets (SupplementaryInfo_1_SpeciesSubsets).

### 2.2 Promoter sequences

Promoter sequences were retrieved from the Eukaryotic Promoter Database (EPD; https://epd.expasy.org/epd) on the 23rd of August 2022 (Cavin Périer et al., 1998). Human promoter sequences were used as reference sequences for all mammals, as the human database contains the largest number of mammalian promoter sequences. Human reference promoter data has also been used for lifespan estimation in mammalian species with success in previous studies (Mayne et al., 2019). Sequences were extracted from −499 to 100 base pairs around the transcription start site of each gene, following the protocol of previous studies (Budd et al., 2023; Mayne et al., 2019). The database had a total of 29,598 promoters for the human reference genome (hg19; SupplementaryInfo_8_hg19_ZLwnj). Orthologous promoter sequences for each species genome were identified using the Basic Local Alignment Search Tool (BLAST) v2.7.1 using default settings (outlined in SupplementaryInfo_2_MetadataASMScript) with percent identity of >70%. If a promoter did not have a BLAST hit the CpG density was recorded as 0.

### 2.3 Sexual maturity predictive model

All analyses were performed in R version 4.2.1 and the custom R script used in this study is available in the supplementary information (R Studio Team, 2015; SupplementaryInfo_2_MetadataASMScript). CpG density was calculated by counting the total number of CpGs in the BLAST hit and dividing it by the sequence length. Pre‐filtering was performed to remove promoters that were identified in less than 90% of species. Species that had <50% of remaining promoters were also removed. An elastic net regression using the glmnet R package was used to identify the minimum number of promoters required for age at sexual maturity prediction (Friedman et al., 2010). A tenfold cross validation was used to estimate the optimal lambda parameter for the model, with the family set to Gaussian, nfolds to 10, nlambda as 100 and alpha as 0.2. A further 500‐fold cross validation was applied to the model to determine the optimal seed value. Species were randomly split into either a training or testing data set with a 60/40% split, respectively. The performance of the model was assessed using Pearson correlations for training and testing datasets and Welch's two‐sample t‐tests for assessing absolute error rates between testing and training sample predictions.

### 2.4 Gene ontology assessments

Enrichr (Chen et al., 2013) was utilized for gene ontology (GO) enrichment analysis using 2018 terms for molecular function, cellular component and biological process. Significant GO results were identified where the adjusted *p*‐value was <0.05. Plots of significant GO terms were generated with ggplot2 (Villanueva & Chen, 2019), using a ‐log_10_ adjusted *p*‐value scale.

## 3 RESULTS

### 3.1 Sexual maturity data set

A total of 93 mammalian species with both genomes and sexual maturity information were identified (SupplementaryInfo_1_SpeciesSubsets). The estimated ASM range for these species was between 28 and 4745 days for females and 41 and 5110 days for males. Sexual maturity information was available for all 93 species in females and 68 species in males. Lifespan information was available for 90 species and enabled the generation of a sexual maturity to lifespan ratios for 90 females and 67 males. The Bornean orangutan (*Pongo pygmaeus*) and Short‐beaked echidna (*Tachyglossus aculeatus*) were removed during pre‐filtering because less than 50% of the *Homo sapiens*’ promoters were identified by BLAST in those species, resulting in CpG density values of 0 for those promoters. Further assessment of the dataset revealed that both monotreme species assessed, the short‐beaked echidna and the platypus (*Ornithorhynchus anatinus*), had relatively high levels of promoters without significant homology to human reference promoters (48.5% and 56.0%, respectively, in males, 42.5% and 50.6% in females), implying a smaller number of orthologous gene promoters could be successfully identified in their genomes compared to other species (SupplementaryInfo_3_PercentageNonZeroPromoterHits). Conversely, more closely related species to *Homo sapiens* had lower proportions of promoters without homology to human reference promoters, with primates such as the common chimpanzee (*Pan troglodytes*), rhesus macaque (*Macaca mulatta*) and gelada baboon (*Theropithecus gelada*) all sharing 100% of the promoter regions with the human set (SupplementaryInfo_3_PercentageNonZeroPromoterHits).

### 3.2 Age at sexual maturity prediction

Utilizing the predictive modelling methods described above, an elastic net regression model was generated for each of the four data subsets, with gene promoters predictive of sexual maturity identified in training datasets and then tested upon the testing datasets. Of the four datasets, both the male ratio and female ratio subsets had *R* ^2^ values of <0.7 and were, therefore, not investigated further (Table 1; SupplementaryInfo_4_ASMModelResults).

**TABLE 1:** Age at sexual maturity predictive model results for all four data subsets.

Model	Training or testing	Total samples	Significance	*R* ^2^
Male	Training	42	<0.001	0.932
	Testing	24	<0.001	0.758
Female	Training	56	<0.001	0.935
	Testing	35	<0.001	0.881
Male Ratio	Training	41	<0.001	0.624
	Testing	24	<0.001	0.431
Female Ratio	Training	56	<0.001	0.767
	Testing	32	<0.001	0.565

*Note*: The reported values are derived from the Pearson correlation between the AnAge sexual maturity values and the model's predicted value.

### 3.3 Male ASM model

**FIGURE 1:**
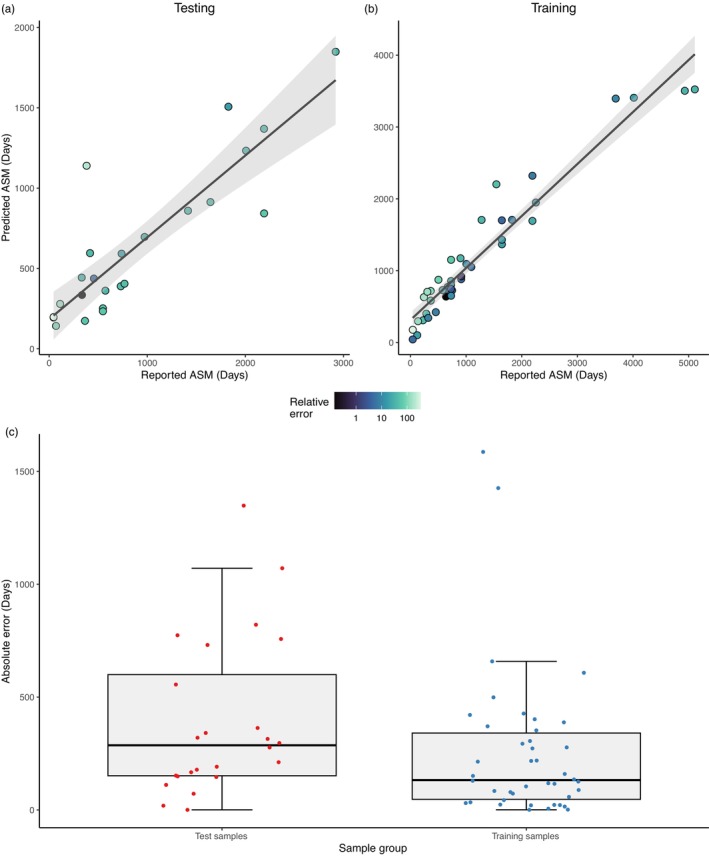
Male age at sexual maturity predictive model result. (a) Correlation plot of male testing dataset. (b) Correlation plot of male training dataset. Relative error is calculated as the absolute error for each predicted ASM and represented as a percentage scale below a and b. (c) Boxplots of absolute error for male testing and training datasets.

For the male subset, the final predictive model utilized 57 promoters (SupplementaryInfo_5_UtilisedGenePromoters) and resulted in significant correlations for both training and testing datasets, with an *R* ^2^ value of 0.932 in the training dataset (Degrees of Freedom (df) = 40, Significance (*p*) < 0.001) and 0.758 in the test dataset (df = 22, *p* < 0.001; Figure 1; Table 1).

The median absolute error for the training and testing datasets was 132 and 286 days, respectively, while the mean absolute error was 252 and 390 days. The median and mean relative error for the training dataset was 16.2% and 37.3%, respectively, while in the testing dataset it was 43.6% and 75.8%, respectively (SupplementaryInfo_4_ASMModelResults). No significant difference was found between the absolute error rates between testing and training data (*p* = 0.123; Figure 1; SupplementaryInfo_4_ASMModelResults), indicating the absence of overfitting.

### 3.4 Female ASM model

**FIGURE 2:**
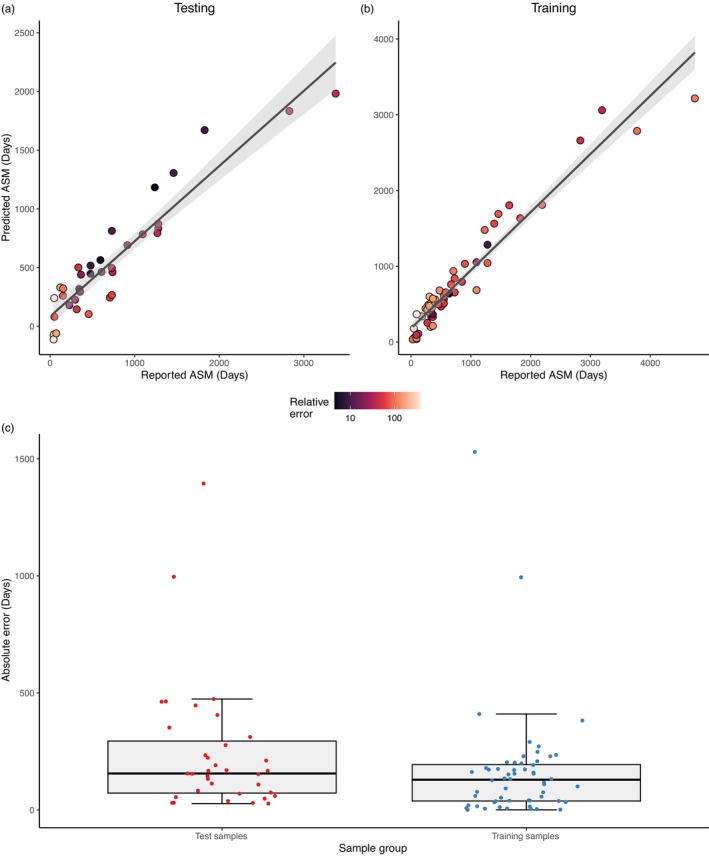
Female age at sexual maturity predictive model results. (a) Correlation plot of female testing dataset. (b) Correlation plot of female training dataset. Relative error is calculated as the absolute error for each predicted ASM and represented as a percentage scale below a and b. (c) Boxplots of absolute error for female testing and training datasets.

For the female subset, a significant correlation was also identified in our final predictive model for both datasets, with an *R* ^2^ value of 0.935 in the training dataset (df = 54, *p* < 0.001) and 0.881 in the test dataset (df = 33, *p* < 0.001; Figure 2; Table 1). This model utilized 71 promoter regions (SupplementaryInfo_5_UtilisedGenePromoters).

For the female predictive model, the median absolute error for the training and testing datasets was 129 and 155 days, respectively, while the mean absolute error was 163 and 242 days. The percentage median and mean relative error was 15.4% and 35.0%, respectively, for the training dataset, while in the testing dataset it was 34.9% and 68.9%, respectively (SupplementaryInfo_4_ASMModelResults). Again, no significant difference was found between the median absolute error rates between training and testing groups which indicates no overfitting occurred (*p* = 0.170; Figure 2; SupplementaryInfo_4_ASMModelResults).

### 3.5 Gene ontology

**FIGURE 3:**
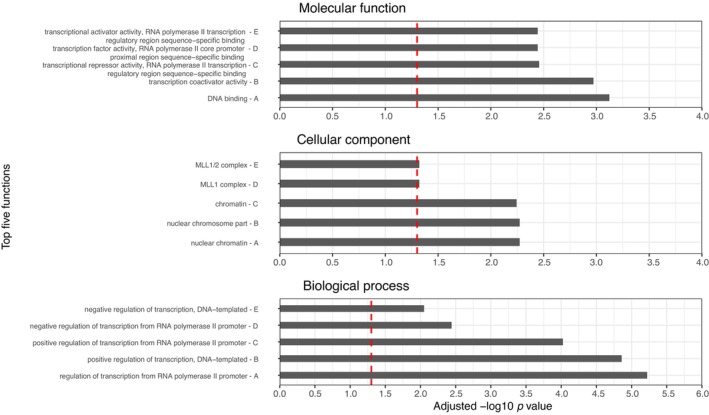
Male gene ontology plots. Plots of top 5 most significantly enriched GO terms for molecular function, cellular component and biological process in the male model. Red dashed line indicates adjusted *p*‐value = 0.05.

**FIGURE 4:**
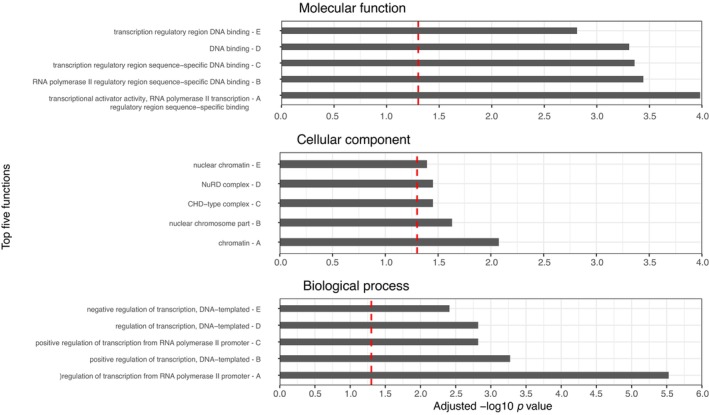
Female gene ontology plots. Plots of top 5 most significantly enriched GO terms for molecular function, cellular component and biological process in the female model. Red dashed line indicates adjusted *p* value = 0.05.

Gene ontology analysis was performed for promoters enriched in each model using the associated gene names from the EPD to identify common characteristics of function. Significant biological processes, cellular components and molecular functional enrichments were identified for both male and female models (SupplementaryInfo_6_GOSignificantResults; Figures 3 and 4). Enriched functions were predominately related to RNA polymerase activities and complexes, regulation of transcription and transcription factor binding, and epigenetic processes such as chromatin binding and histone complexes.

For the male model promoters, the top five significant biological processes all related to the regulation of transcription, either DNA templated or from RNA polymerase II promoters (Figure 3). For cellular components, chromatin or nuclear chromatin comprised the top three significantly enriched components, with the other two being related to the MLL1 complex, a complex of proteins involved in epigenetic regulation, specifically through the core component, histone methyltransferase enzyme (Avdic et al., 2011). Finally, the top five significant molecular functions all related to transcriptional activators, repressors and factor activity, as well as DNA binding.

Similar patterns were found for the female modelled promoters, with very similar biological processes and molecular functions identified compared to the male set (Figure 4; SupplementaryInfo_6_GOSignificantResults). This was also largely true for the cellular components; however, instead of MLL1 complexes, the NuRD and CHD‐type complexes were present in the top five significant enrichments. Both of these complexes are involved with chromatin remodelling and gene regulation, contributing to the precise control of gene expression by modifying the structure of chromatin, influencing the accessibility of regulatory elements and thereby regulating various biological processes during development and differentiation (Denslow & Wade, 2007; Marfella & Imbalzano, 2007). These all signify a strong association between the modelled gene promoters and epigenetic processes.

## 4 DISCUSSION

We developed a genomic approach for estimating species‐level ASM. This approach follows studies that have generated predictions of species lifespan from analysis of CpG densities at promoters selected from a genome‐wide scan to provide estimates of these key life history traits (Budd et al., 2023; Mayne et al., 2020). Our predictive models demonstrated strong correlations between predicted and AnAge ASM values for both female and male mammals. The female model exhibited a coefficient of determination (*R* ^2^) of 0.881 in the test dataset, indicating that 88.1% of the variation between AnAge ASM and predicted female ASM could be explained by CpG density in gene promoter regions. The male model performed similarly, explaining 75.8% of the variation. Higher *R* ^2^ values for the female dataset may be due to a larger sample size or an indication of a higher fidelity input dataset from the AnAge dataset, as female ASM is generally easier to ascertain, with male values likely not being as precise or potentially duplicated from the female value (Geissmann, 1991; Lanyon & Burgess, 2019). Both ratio models performed poorly (*R* ^2^ of 0.431 for males and 0.565 for females), indicating that lifespan did not appear to be an effective proxy to determine the ASM of a mammal in this context. This, therefore, supported the use of specific ASM calibration data for a genomic ASM predictor and that current models for lifespan may not be easily adaptable for ASM estimation. The absence of correlation between the sexual maturity to lifespan ratio and CpG promoter regions suggests that additional mechanisms influence this relationship. Reproductive senescence, particularly in mammals, aligns with the grandmother hypothesis (Nattrass et al., 2019), wherein non‐reproductive older females contribute to the care of younger individuals. Moreover, variations in the onset of puberty, driven by diverse evolutionary adaptations, likely contribute to the high variability observed in the sexual maturity to lifespan ratios across this group. Further research should assess specific causal mechanisms underlying these observations, providing a greater understanding of the relationships between epigenetic regulation, life history traits and evolutionary adaptations.

Gene ontology analysis suggested that the promoters utilized in our models belonged to genes involved in a range of growth and gene regulatory functions (SupplementaryInfo_5_UtilisedGenePromoters; SupplementaryInfo_7_OverlappingGenesByModel). Twenty‐seven of the genes were found in both male and female models and were associated with transcriptional regulation, DNA methylation, muscle growth regulation and developmental processes (Haberland et al., 2007; Laget et al., 2010; Simeone, 1998; Stinckens et al., 2008). The presence of these gene promoters in both models also indicates their potential as biomarkers for sexual maturity age which may lead to the development of targeted assays that don't require full genome analysis. For example, DNA methylation patterns or gene expression levels of these genes could serve as useful biomarkers for individual sexual maturity status in mammalian populations. The development of methylation‐specific PCR or qPCR assays would allow for rapid sample assessment and eliminate the requirement for whole‐genome sequence data. In addition to providing useful information for biomarker development, the gene ontology enrichment analysis of the promoter regions revealed enrichments related to RNA polymerase activities, regulation of transcription, transcription factor binding and other epigenetic processes such as chromatin binding and histone methylation (SupplementaryInfo_6_GOSignificantResults). While epigenetic mechanisms are known to be involved in the regulation of sex and sexual maturity, at present these specific pathways and the modifications involved are not fully characterized (Nagahama et al., 2021). Our findings contribute to our increasing knowledge of this process and suggest that other epigenetic mechanisms, in addition to DNA methylation, warrant further research.

Despite the apparently high statistical performance of the models presented in this study, they are likely to have several limitations resulting from the data they are based on. Firstly, the ASM dataset from the AnAge database is the most comprehensive but may not be the most accurate (De Magalhães & Costa, 2009). Furthermore, the sample size of mammalian species used in the analysis was also constrained by the availability of complete chromosome level genome assemblies. To overcome these limitations, future research should aim to incorporate more comprehensive genomic and diverse age at maturity datasets to validate and refine the models. Using additional genome repositories would also be beneficial. This study shows the potential use of CpG density as a predictor of ASM, but more work is required to improve the accuracy of the predictions. In the testing dataset, the median relative error rates were 34.9% for females and 43.6% for males, indicating a high level of deviation. These error rates are comparable to those recently reported for CpG density derived predictions of lifespan in fish, which reported 36.8% median relative error rates (Budd et al., 2023). Refinement of the model, potentially to more closely related species groups or larger sample sizes, may reduce this relative error value. The observation that more closely related species to the EPD reference species (*Homo sapiens*) had lower proportions of 0% density promoters (largely resulting from the absence of a BLAST hit) highlights that the species used to identify orthologous promoters in other species may limit promoter identification in distantly related species (Ferguson‐Smith & Trifonov, 2007). Therefore, modelling a smaller taxonomic group using closely related species as the reference promoter set may result in higher orthologous promoter identifications and potentially increased predictive power. However, there would likely be a substantial trade‐off between potential increases in model performance gained by restricting the dataset to a closely related group and the decreased statistical power associated with a reduction in sample size. Additionally, while our model focussed on mammals, this was primarily because mammals have the largest number of publicly available genomes and would, therefore, allow for a taxonomically restricted dataset but with a high number of individual species with closely related gene promoter regions. Broader scale models could also be created, though potentially with reduced accuracy due to genomic divergence in gene promoters (Mayne et al., 2019). Adapting these models to other taxonomic groups using the relevant EPD reference species is likely to result in more accurate predictions (Budd et al., 2023) and is a key area for future work.

The potential practical implications of this research are substantial, particularly for population biology and conservation management. Accurate estimation of ASM is crucial for assessing population dynamics, setting conservation goals and making informed management decisions (Heydenrych et al., 2021; Lanyon & Burgess, 2019). The non‐invasive nature of our models, relying solely on genomic data, which is becoming increasingly affordable to generate, makes them valuable tools for assessing populations without the need for invasive sampling procedures. Furthermore, the ability to provide rapid estimates of species ASM in the absence of long‐term monitoring or large‐scale sampling efforts substantially increases the feasibility of genomic ASM prediction over previously existing methods. Our study also further strengthens the link between epigenetic processes and life history traits, emphasizing the potential of CpG density or other epigenetic or RNA‐based analyses as predictive biomarkers. Previous studies have demonstrated the relationship between genomic features linked to DNA methylation patterns, specifically CpG density in gene promoter regions, and various life history traits, including lifespan (Budd et al., 2023; Heydenrych et al., 2021; Mayne et al., 2020; McLain & Faulk, 2018; Tamagawa et al., 2017). By extending this approach to sexual maturity age estimation, our research highlights the broader utility of epigenetic biomarkers in understanding and predicting key life history traits.

## 5 CONCLUSION

The models in this study demonstrate strong performance for prediction of ASM in female and male mammals, offering valuable tools for population biology, conservation management and historical estimations. The identification of specific gene promoter regions associated with species‐level ASM provide potential targets for genetic or epigenetic ASM biomarkers that could be used for determining individual sexual maturity status. Future research efforts in this area should focus on expanding datasets and validating the models across a more diverse range of species and taxonomic groups. Overall, this study expands upon the rapidly growing field of epigenomic tools for conservation and population biology, providing valuable insights into sexual maturity and supporting effective management strategies.

## CONFLICT OF INTEREST STATEMENT

The authors have no conflicts of interest to declare.

## Supporting information


Appendix S1.

Appendix S2.

Appendix S3.

Appendix S4.

Appendix S5.

Appendix S6.

Appendix S7.

Appendix S8.

Appendix S9.

Appendix S10.

Appendix S11.

Appendix S12.

Data S1.

## Data Availability

Data sharing is not applicable to this article as no new data were created or analysed in this study. The R script used in this study is provided in the supplementary information (SupplementaryInfo_2_MetadataASMScript) as is the *Homo sapiens* promoter regions file (SupplementaryInfo_8_hg19_ZLwnj).
