# Supplementary material for: A genomic predictor for age at sexual maturity for mammalian species
Source: Evol Appl. 2024 Jan 10;17(2):e13635. doi: 10.1111/eva.13635 (PMC10853647; doi:10.1111/eva.13635)
Supplement: Supplementary file 2 — Appendix S2. [file EVA-17-e13635-s012.docx]

##########################################

### Sexual Maturity model using GLMNET ###

##########################################

### Needed files : HG19 promoter file in csv format - Hit files for blasts - pd_XXX file for sex mat ages ###

### Below is example Blast command for the Blast files ###

#echo 'Step 3: Run Blastn against the known promoter sequences'

#blastn -query $PROMOTERS -db $WD_DIR/$l -out $BLASTN_DIR/$m -outfmt "6 qseqid sseqid slen #qstart qend sstart send pident nident sseq" -perc_identity 70 -culling_limit 1

### Must change the seednum for best seed, and dataset name ###

### Built on R 4.2.1 ###

# Load libraries

library**(**tidyverse**)** #Graphs and visualising

library**(**devtools**)** #Needed for mosaic

library**(**dplyr**)** #For some renaming functions

library**(**ggrepel**)** #Needed for plots, must install the ggrepel package

library**(**tweenr**)** #Needed for mosaic install.packages("tweenr")

library**(**mosaic**)** #Graphs and visualising - Need to install packages - devtools

library**(**Biostrings**)** # Finding CG sequences - Need install.packages("BiocManager")

library**(**data.table**)** # Required for merging data tables

library**(**caret**)** # Used to split data

library**(**glmnet**)** # Contains the elastic net regression model

library**(**Metrics**)** #For calculating relative absolute error

library**(**tibble**)** #For converting rownames to columns

library**(**broom**)** #For putting t test results into dataframe

library**(**enrichR**)** #Load enrichR package for GO analysis

library**(**RColorBrewer**)** #Nice colour palettes for graphs

library**(**viridis**)** #Used for colourblind friendly colourschemes

palette **<-** brewer.pal**(**8, "Set1"**)** #Sets the palette to Set1

### Check below and change if needed ###

#############################

### Editable Values Begin ###

#############################

### Set which dataset is being used - Set to either Female, Male, FemaleRatio, or MaleRatio

# Make sure to wipe the environment between runs to ensure no issues

# Also set the seed to the appropriate seed for the dataset

DatasetUsed **<-** "Male"

### Set whether iteration is needed

# If you need to run the iteration for seed num then change the below to 1, otherwise set as 2

RunIteration **=** 2

# Number of iterations to complete (500 works well for filtered file)

NumIterations **=** 500

### Set seed number for the main model

# Set appropriate to dataset used, or based on datITERATION, use 1 if not run prior

# Best Seeds - Male 400, MaleRatio 156, Female 435, FemaleRatio 352

SeedNum **=** 400

### Sets the split of training versus testing samples, lower with less samples available

# 0.6 is current split, due to low sample number but needing enough for test set (40%)

SplitPer **=** 0.6

### Sets the Alpha value to the desired value for glmnet modelling

# Between 0 and 1 is elastic net regression

# Towards 1 is more restrictive in # of promoters used (more lasso, less ridge) - 0 is ridge, 1 is lasso

# 0.2 is more towards ridge, indicating more promoters may be used with less of an input each

AlphaVal **=** 0.2

### Sets the filter percentage for promoter rows - 90% is 0.9 and works well

# Set to 0 for no filter, but column filters is more restrictive then

RowFilter **=** 0.9

### Sets the filter percentage for species cols - 50% is 0.5 and works well with 0.9 RowFilter

# 0.5 filters out poor qual pongo and echidna (monotreme) - Only if RowFIlter is 0.9

# 0.7 Takes out both monotremes (echidna and platypus) and pongo - Only if RowFIlter is 0.9

ColFilter **=** 0.5

###########################

### Editable Values End ###

###########################

### Setting colour dependent on data set used

**if** **(**DatasetUsed **==** "Male" **||** DatasetUsed **==** "MaleRatio"**)** **{**

PlotColour **<-** "blue"

ViridisSetting **<-** "G"

**}** **else** **{**

PlotColour **<-** "red"

ViridisSetting **<-** "F"

**}**

### Read in one blast hit file to kick off dataframe with CpG density

setwd**(**paste0**(**"G:/My Drive/Meta_AnalysisWork/DATAAUG22/", DatasetUsed, "/"**))**

### Below is used for file output naming

wdinput **<-** paste0**(**"G:/My Drive/Meta_AnalysisWork/DATAAUG22/", DatasetUsed, "/"**)**

wdcurrent **<-** dirname**(**rstudioapi**::**getSourceEditorContext**()$**path**)**

### Get the list of blast hit files to import into R - Imports text files which have hits of promoters

datFILES **<-** list.files**(**path **=** paste0**(**"G:/My Drive/Meta_AnalysisWork/DATAAUG22/", DatasetUsed, "/"**)**, pattern **=** "_hits.txt"**)**

### Move table into where the files are kept

datTMP **<-** read.table**(**file **=** datFILES**[**1**])**

### Remove duplicates

datTMP **<-** datTMP**[!**duplicated**(**datTMP**[**,1**])**, **]**

### Add in two columns with blast length and CpG frequency

datTMP**$**Length **<-** "NA"

datTMP**$**CG_Frequency **<-** "NA"

datTMP**$**CG_Density **<-** "NA"

### Initial loop to get CpG density for the first file - Makes sure to remove dashes in blast hits files

**for(**i **in** 1**:**nrow**(**datTMP**)){**

datTMP**$**Length**[**i**]** **<-** nchar**(**gsub**(**"-", "", as.character**(**datTMP**[**i,10**])))**

datTMP**$**CG_Frequency**[**i**]** **<-** dinucleotideFrequency**(**DNAString**(**datTMP**[**i,10**]))[**"CG"**]**

datTMP**$**CG_Density**[**i**]** **<-** as.numeric**(**datTMP**$**CG_Frequency**[**i**])** **/** as.numeric**(**datTMP**$**Length**[**i**])**

**}**

### Change datTMP to the final data table

colnames**(**datTMP**)[**ncol**(**datTMP**)]** **<-** gsub**(**pattern **=** "_hits.txt", replacement **=** "", datFILES**[**1**])**

colnames**(**datTMP**)[**1**]** **<-** "Promoter_ID"

### datDENSITY will contain the CpG density for all the species

datDENSITY **<-** data.table**(**datTMP**[**c**(**1,ncol**(**datTMP**))]**, key **=** colnames**(**datTMP**[** ,c**(**1,ncol**(**datTMP**))]))**

### Next loop to read in all the species ###

##################

### Loop Start ###

##################

### Progress bar ###

ProgressBar **<-** winProgressBar**(**title **=** "Progress Bar", min **=** 0, max **=** **(**length**(**datFILES**)** **-** 1**)**, width **=** 300**)**

**for(**i **in** 2**:**length**(**datFILES**)){**

Sys.sleep**(**0.1**)** # Part of progress bar

### Print the name of the sample being processed ###

### Read in the file

datTMP **<-** read.table**(**file **=** datFILES**[**i**])**

### Remove duplicates

datTMP **<-** datTMP**[!**duplicated**(**datTMP**[**,1**])**, **]**

### Add in two columns with blast length and CpG frequency

datTMP**$**Length **<-** "NA"

datTMP**$**CG_Frequency **<-** "NA"

datTMP**$**CG_Density **<-** "NA"

**for(**j **in** 1**:**nrow**(**datTMP**)){**

datTMP**$**Length**[**j**]** **<-** nchar**(**gsub**(**"-", "", as.character**(**datTMP**[**j,10**])))**

datTMP**$**CG_Frequency**[**j**]** **<-** dinucleotideFrequency**(**DNAString**(**datTMP**[**j,10**]))[**"CG"**]**

datTMP**$**CG_Density**[**j**]** **<-** as.numeric**(**datTMP**$**CG_Frequency**[**j**])** **/** as.numeric**(**datTMP**$**Length**[**j**])**

**}**

### Change datTMP to the final data table

colnames**(**datTMP**)[**ncol**(**datTMP**)]** **<-** gsub**(**pattern **=** "_hits.txt", replacement **=** "", datFILES**[**i**])**

colnames**(**datTMP**)[**1**]** **<-** "Promoter_ID"

datTMP **<-** data.table**(**datTMP**[**c**(**1,ncol**(**datTMP**))]**, key **=** colnames**(**datTMP**[** ,c**(**1,ncol**(**datTMP**))]))**

### Merge datDENSITY with datTMP

datDENSITY **<-** merge**(**x **=** datDENSITY, y **=** datTMP, by **=** "Promoter_ID", all **=** **TRUE)**

### Update the progress bar

setWinProgressBar**(**ProgressBar, i, title**=**paste**(**round**(**i**/(**length**(**datFILES**)** **-** 1**)** ***** 100, 0**)**, "% Completed"**))**

**}**

### Close the progress bar

close**(**ProgressBar**)**

setwd**(**"~"**)**

###################

### End of loop ###

###################

### Quick Clean up

rm**(**datTMP, ProgressBar, datFILES, i, j**)**

### Reorders datDENSITY file and makes it a df

datDENSITY **<-** data.frame**(**datDENSITY**)**

row.names**(**datDENSITY**)** **<-** datDENSITY**$**Promoter_ID

datDENSITY **<-** datDENSITY**[** ,**-**1**]**

### Read in the meta data file - One column is Species (separated with _), the other is Maturity

pd **<-** read.csv**(**paste0**(**wdinput, "pd_", DatasetUsed, ".csv"**))**

### Prefiltering steps for datDENSITY ###

### Changes NA values to zeros

datDENSITY**[**is.na**(**datDENSITY**)]** **<-** 0

### Code to filter out mostly zero promoter hits - These form false data if left

# Calculate percentage of non-zero values for each row

percent_non_zero_rows **<-** apply**(**datDENSITY, 1, **function(**x**)** sum**(**x **!=** 0**)** **/** length**(**x**))**

### Filter rows with more than XX% non-zero values, as these values will skew results

datDENSITYfiltered **<-** datDENSITY**[**percent_non_zero_rows **>** RowFilter, **]**

### Calculate percentage of non-zero values for each column

percent_non_zero_col **<-** apply**(**datDENSITYfiltered, 2, **function(**x**)** sum**(**x **!=** 0**)** **/** length**(**x**))**

### Filter columns with less than XX% non-zero values (0.7 takes out monotremes, 0.5 takes out poor qual pongo)

FilteredInSpecies **<-** datDENSITYfiltered**[**, percent_non_zero_col **>** ColFilter**]**

FilteredOutSpecies **<-** datDENSITYfiltered**[**, percent_non_zero_col **<** ColFilter**]**

### Makes a df of Percent non_zero columns (promoters) post initial row filtering and saves a csv for reference

Percent_NonZero_PostFilter **<-** as.data.frame**(**percent_non_zero_col**)**

write.csv**(**Percent_NonZero_PostFilter, file **=** paste0**(**wdinput, "SpeciesPromoterPercentages", DatasetUsed, ".csv"**)**, row.names **=** **TRUE)**

### New datDENSITYfiltered file which only includes species which have 50% of the same promoter hits as other species

# Can comment edit this line out to include all mammal species (MONOTREMES drop at 70%, other marsupials at 80)

datDENSITYfiltered **<-** FilteredInSpecies

### Save the filtered in and out species

InColumnNames **<-** colnames**(**FilteredInSpecies**)**

write.csv**(**data.frame**(**InColumnNames**)**, file **=** paste0**(**wdinput, "FilteredInSpecies", DatasetUsed, ".csv"**)**, row.names **=** **FALSE)**

OutColumnNames **<-** colnames**(**FilteredOutSpecies**)**

write.csv**(**data.frame**(**OutColumnNames**)**, file **=** paste0**(**wdinput, "FilteredOutSpecies", DatasetUsed, ".csv"**)**, row.names **=** **FALSE)**

### Check if the order of species match up between data frames

### Reorder pd to match the species order in datDENSITYfiltered

pd **<-** pd**[**match**(**colnames**(**datDENSITYfiltered**)**, pd**$**Species**)**,**]**

all**(**colnames**(**datDENSITYfiltered**)** **==** pd**$**Species**)**

### Transpose for elastic net to work with

datDENSITYfiltered **<-** data.frame**(**t**(**datDENSITYfiltered**))**

### Natural log transform sexual maturity age to fit a linear model - Only need this when doing log calculations

pd**$**Maturity_Log **<-** log**(**pd**$**Maturity**)**

### Plot the percentage of zero hits in the promoter density unfiltered file

### Calculate percentage of zero values for each column

percent_zero_col **<-** colMeans**(**datDENSITY **==** 0**)** ***** 100

### Sort the percentage of zero values and get the corresponding order

order_indices **<-** order**(**percent_zero_col, decreasing **=** **TRUE)**

### Reorder the percentage of zero values and the x-axis labels based on the order

percent_zero_col_reordered **<-** percent_zero_col**[**order_indices**]**

labels_reordered **<-** colnames**(**datDENSITY**)[**order_indices**]**

### Create a data frame for the plot

dfPerZero **<-** data.frame**(**Species **=** factor**(**labels_reordered, levels **=** labels_reordered**)**, Percentage **=** percent_zero_col_reordered**)**

### Create the ggplot object with ordered factor levels

ggplot**(**dfPerZero, aes**(**x **=** Species, y **=** Percentage**))** **+**

geom_bar**(**stat **=** "identity", width **=** 0.8**)** **+**

coord_flip**()** **+**

labs**(**title **=** "Percentage of Zero Values by Species",

x **=** "Species",

y **=** "Percentage"**)** **+**

ylim**(**0, 100**)** **+**

theme**(**axis.text.x **=** element_text**(**angle **=** 90, hjust **=** 1, vjust **=** 0.5, size **=** 6**))**

### Save the data frame as a CSV file

write.csv**(**dfPerZero, paste0**(**wdinput, "PercentZeroPromoterHits", DatasetUsed, ".csv"**)**, row.names **=** **FALSE)**

########################

### Model Generation ###

########################

######################

### Iteration Code ###

######################

### Script to run a loop of different seed values, to identify seeds with minimal var

### RUN THE BELOW ONLY WHEN REQUIRED - Takes a long time to run (less if pre-filtered)

### Can change the 3 following 1-XXX to either 1:50 or 1:500 depending on time requirements

### Create dataframe, with Iterations (Determined above), with output columns of meaningful statistics for each seed number

**if** **(**RunIteration **==** 1**)**

**{**

datITERATION **<-** data.frame**(**Iteration **=** 1**:**NumIterations, TrainingCor **=** **NA**, TestingCor **=** **NA**, TrainingMedAbsErr **=** **NA**, TestingMedAbsErr **=** **NA)**

**for(**i **in** 1**:**NumIterations**){**

print**(**i**)** #Shows a running print of which i number it has reached

### Split the data into either a training or testing data set - separates within quantiles already

set.seed**(**i**)** #Using the i number for each loop

sampleINDEX **<-** createDataPartition**(**y **=** pd**$**Maturity, times **=** 1, p **=** SplitPer, list **=** **FALSE)** # Change to p to 0.7 for 70/30 split

### Split the CpG density data into a training and testing data set

datTRAIN **<-** data.matrix**(**datDENSITYfiltered**[**sampleINDEX, **])**

datTEST **<-** data.matrix**(**datDENSITYfiltered**[-**sampleINDEX, **])**

### Split the phenotype data

pdTRAIN **<-** pd**[**sampleINDEX, **]**

pdTEST **<-** pd**[-**sampleINDEX, **]**

### Use a 10 fold cross validation to estimate the lambda parameter (Training data)

set.seed**(**i**)**

glmnet.Training.CV **<-** cv.glmnet**(**x **=** datTRAIN, y **=** pdTRAIN**$**Maturity, nfolds**=**10, family**=**"gaussian", alpha **=** AlphaVal**)**

### The definition of the lambda parameter:

lambda.glmnet.Training **<-** glmnet.Training.CV**$**lambda.min # May need to record in manuscript

### Fit the elastic net predictor to the training data

glmnet.Training **<-** glmnet**(**datTRAIN, pdTRAIN**$**Maturity, family**=**"gaussian", nlambda**=**100, alpha **=** AlphaVal, intercept **=** **FALSE)**

### Estimate the sexual maturity in the training data set

datTrainPredict **<-** predict**(**glmnet.Training, datTRAIN, type**=**"response", s**=**lambda.glmnet.Training**)**

### Extract the promoters that are predictive of sexual maturity

datPROMOTERS **<-** as.matrix**(**coef**(**glmnet.Training, s**=**lambda.glmnet.Training**))**

datPROMOTERS **<-** as.matrix**(**datPROMOTERS**[**which**(**datPROMOTERS**[**,1**]** **>** 0 **|** datPROMOTERS**[**,1**]** **<** 0**)**,**])**

### datPROMOTERS is the model

### Estimate the age of sexual maturity in the testing data set

datTestPredict **<-** predict**(**glmnet.Training, datTEST, type**=**"response", s**=**lambda.glmnet.Training**)**

### Training Correlation

datITERATION**$**TrainingCor**[**i**]** **<-** cor.test**(**x **=** **(**pdTRAIN**$**Maturity**)**, y **=** **(**datTrainPredict**[**,1**]))$**estimate

### Testing Correlation

datITERATION**$**TestingCor**[**i**]** **<-** cor.test**(**x **=** **(**pdTEST**$**Maturity**)**, y **=** **(**datTestPredict**[**,1**]))$**estimate

### Training Median Absolute error

datITERATION**$**TrainingMedAbsErr**[**i**]** **<-** median**(**abs**(**pdTRAIN**$**Maturity **-** **(**datTrainPredict**[**,1**])))**

### Testing Median Absolute error

datITERATION**$**TestingMedAbsErr**[**i**]** **<-** median**(**abs**(**pdTEST**$**Maturity **-** **(**datTestPredict**[**,1**])))**

### Testing Mean Absolute error

datITERATION**$**TestingMeanAbsErr**[**i**]** **<-** mean**(**abs**(**pdTEST**$**Maturity **-** **(**datTestPredict**[**,1**])))**

### Difference between training and testing abs err

datITERATION**$**MedAbsErrDiff**[**i**]** **<-** sum**(**datITERATION**$**TrainingMedAbsErr**[**i**]** **-** datITERATION**$**TestingMedAbsErr**[**i**])**

### Median and mean relative error as a percentage

datITERATION**$**MedRelPercErrTest**[**i**]** **<-** median**(**abs**((**datTestPredict **-** pdTEST**$**Maturity**)/**pdTEST**$**Maturity *****100**))**

datITERATION**$**MeanRelPercErrTest**[**i**]** **<-** mean**(**abs**((**datTestPredict **-** pdTEST**$**Maturity**)/**pdTEST**$**Maturity *****100**))**

### Ttest of differnce in Absolute Error to test for overfit, and stored for dataframe to print

IterTTestResult **=** as.data.frame**(**tidy**(**t.test**(**x **=** abs**(**datTrainPredict **-** pdTRAIN**$**Maturity**)**, y **=** abs**(**datTestPredict **-** pdTEST**$**Maturity**)**, paired **=** F**)))**

datITERATION**$**AbsErrTTestSig**[**i**]** **<-** IterTTestResult**$**p.value

**}**

### Writes the full iteration results to csv

write.csv**(**datITERATION, paste0**(**wdinput, "IterationSeedResult", DatasetUsed, ".csv"**)**, row.names **=** **FALSE)**

**}**

### Use above for the best SEED for below

###################

### FINAL MODEL ###

###################

### Split the data into either a training or testing data set - CAN change maturity to Maturity_Log, worse models though

set.seed**(**SeedNum**)**

sampleINDEX **<-** createDataPartition**(**y **=** pd**$**Maturity, times **=** 1, p **=** SplitPer, list **=** **FALSE)** # Change to p to 0.7 for 70/30 split

### Split the CpG density data into a training and testing data set

datTRAIN **<-** data.matrix**(**datDENSITYfiltered**[**sampleINDEX, **])**

datTEST **<-** data.matrix**(**datDENSITYfiltered**[-**sampleINDEX, **])**

### Split the SexMat dataset into training and testing portions

pdTRAIN **<-** pd**[**sampleINDEX, **]**

pdTEST **<-** pd**[-**sampleINDEX, **]**

### Use a 10 fold cross validation to estimate the lambda parameter (Training data)

set.seed**(**SeedNum**)**

glmnet.Training.CV **<-** cv.glmnet**(**x **=** datTRAIN, y **=** pdTRAIN**$**Maturity, nfolds**=**10, family**=**"gaussian", alpha **=** AlphaVal**)**

### The definition of the lambda parameter:

lambda.glmnet.Training **<-** glmnet.Training.CV**$**lambda.min # May need to record in manuscript

### Fit the elastic net predictor to the training data - Can change maturity to log

glmnet.Training **<-** glmnet**(**datTRAIN, pdTRAIN**$**Maturity, family**=**"gaussian", nlambda**=**100, alpha **=** AlphaVal, intercept **=** **FALSE)**

### Estimate the sexual maturity in the training data set

datTrainPredict **<-** predict**(**glmnet.Training, datTRAIN, type**=**"response", s**=**lambda.glmnet.Training**)**

### Extract the promoters that are predictive of sexual maturity

datPROMOTERS **<-** as.matrix**(**coef**(**glmnet.Training, s**=**lambda.glmnet.Training**))**

datPROMOTERS **<-** as.matrix**(**datPROMOTERS**[**which**(**datPROMOTERS**[**,1**]** **>** 0 **|** datPROMOTERS**[**,1**]** **<** 0**)**,**])**

### datPROMOTERS is the model ###

### Estimate the age of sexual maturity in the testing data set

datTestPredict **<-** predict**(**glmnet.Training, datTEST, type**=**"response", s**=**lambda.glmnet.Training**)**

##############################################

### Determine the performance of the model ###

##############################################

### Determine the pearson correlation in both the training and testing data set

### Plot the training data - Can remove the exp from in front of dat Predict if not using logged values

# Is just for quick visual use

plot**(**pdTRAIN**$**Maturity, **(**datTrainPredict**))**

cor.test**(**x **=** pdTRAIN**$**Maturity, y **=** **(**datTrainPredict**))**

abline**(**lm**(((**datTrainPredict**))** **~** pdTRAIN**$**Maturity**))**

### Plot the testing data just for internal use. doesn't save

plot**(**pdTEST**$**Maturity, **(**datTestPredict**))**

cor.test**(**x **=** pdTEST**$**Maturity, y **=** **(**datTestPredict**))**

abline**(**lm**(((**datTestPredict**))** **~** pdTEST**$**Maturity**))**

### Change datTrainPredict to data frame, not atomic - Allows for use within following code lines

datTrainPredictX **<-** as.data.frame**(**datTrainPredict**)**

is.atomic**(**datTrainPredictX**)**

### Change datTestPredict to data frame, not atomic

datTestPredictX **<-** as.data.frame**(**datTestPredict**)**

is.atomic**(**datTestPredictX**)**

### Remember, if log transformed for model, all datTestPredicts must have exp added

### Make data frame of training values, both predicted and actual

dataTRAINX **<-** data.frame**(**pred **=** **(**datTrainPredictX**$**s1**)**, actual **=** pdTRAIN**$**Maturity**)**

### Make data frame of testing values, both predicted and actual

dataTESTX **<-** data.frame**(**pred **=** **(**datTestPredictX**$**s1**)**, actual **=** pdTEST**$**Maturity**)**

### Error Metrics ###

### These are used for the final results table

### Assess Mean Absolute Error testing

Model_TestMeanAE **<-** mae**((**datTestPredict**)**,pdTEST**$**Maturity**)**

mae**((**datTestPredict**)**,pdTEST**$**Maturity**)**

### Assess Mean Absolute Error training

Model_TrainMeanAE **<-** mae**((**datTrainPredict**)**,pdTRAIN**$**Maturity**)**

mae**((**datTrainPredict**)**,pdTRAIN**$**Maturity**)**

### Assess Median Absolute Error testing

Model_TestMedAE **<-** mdae**((**datTestPredict**)**,pdTEST**$**Maturity**)**

mdae**((**datTestPredict**)**,pdTEST**$**Maturity**)**

### Assess Median Absolute Error training

Model_TrainMedAE **<-** mdae**((**datTrainPredict**)**,pdTRAIN**$**Maturity**)**

mdae**((**datTrainPredict**)**,pdTRAIN**$**Maturity**)**

### Assess Relative Error test - Gives percentage values

Model_TestRelErr **<-** abs**((**datTestPredict **-** pdTEST**$**Maturity**)/**pdTEST**$**Maturity *****100**)**

median**(**Model_TestRelErr**)**

mean**(**Model_TestRelErr**)**

### Assess Mean Relative Error train

Model_TrainRelErr **<-** abs**((**datTrainPredict **-** pdTRAIN**$**Maturity**)/**pdTRAIN**$**Maturity *****100**)**

median**(**Model_TrainRelErr**)**

mean**(**Model_TrainRelErr**)**

### Add AbsErr column to both the TRAIN and TEST results, also add relative error - This is used for RelErr plots in following sections

dataTRAINX**[**'AbsErr'**]** **=** abs**(**dataTRAINX**$**actual **-** dataTRAINX**$**pred**)**

dataTESTX**[**'AbsErr'**]** **=** abs**(**dataTESTX**$**actual **-** dataTESTX**$**pred**)**

dataTRAINX**[**'RelErr'**]** **=** dataTRAINX**$**AbsErr **/** dataTRAINX**$**actual ***** 100

dataTESTX**[**'RelErr'**]** **=** dataTESTX**$**AbsErr **/** dataTESTX**$**actual ***** 100

### Ttests for AbsErr ###

### Ttest of differnce in Absolute Error to test for overfit, and stored for dataframe to print

TTestResult **=** tidy**(**t.test**(**x **=** abs**(**dataTRAINX**$**pred **-** dataTRAINX**$**actual**)**, y **=** abs**(**dataTESTX**$**pred **-** dataTESTX**$**actual**)**, paired **=** F**))**

XTTestResult **=** as.data.frame**(**TTestResult**)**

### Correlation Final Results ###

### Making dataframes from test results, then appending together

ModelTestResultStats **=** tidy**(**cor.test**(**x **=** pdTEST**$**Maturity, y **=** **(**datTestPredict**)))**

XModelTestResultStats **=** as.data.frame**(**ModelTestResultStats**)**

ModelTrainResultStats **=** tidy**(**cor.test**(**x **=** pdTRAIN**$**Maturity, y **=** **(**datTrainPredict**)))**

XModelTrainResultStats **=** as.data.frame**(**ModelTrainResultStats**)**

FinalModelResults **=** rbind**(**XModelTestResultStats, XModelTrainResultStats**)**

row.names**(**FinalModelResults**)[**1**]** **<-** "TestingResults"

row.names**(**FinalModelResults**)[**2**]** **<-** "TrainingResults"

colnames**(**FinalModelResults**)[**1**]** **<-** "Correlation"

colnames**(**FinalModelResults**)[**2**]** **<-** "t"

colnames**(**FinalModelResults**)[**4**]** **<-** "DF"

### Determine R2 values

R2Test **<-** **(**XModelTestResultStats**[**1**])^**2

R2Train **<-** **(**XModelTrainResultStats**[**1**])^**2

FinalModelResults**[**1,9**]** **=** R2Test

FinalModelResults**[**2,9**]** **=** R2Train

colnames**(**FinalModelResults**)[**9**]** **<-** "RSquaredValues"

### Writes model summary to a CSV file - Changes name to whichever summary file it is for

write.csv**(**XTTestResult, paste0**(**wdinput, "FinalTTestModelResults", DatasetUsed, ".csv"**)**, row.names **=** **FALSE)**

write.csv**(**FinalModelResults, paste0**(**wdinput, "FinalCorrModelResults", DatasetUsed, ".csv"**)**, row.names **=** **FALSE)**

### Summary of All Model Results ###

#### Create a file with results ###

ModelResults **<-** data.frame**(**ChosenSeed **=** **NA**,

TrainingCor **=** **NA**,

TestingCor **=** **NA**,

TrainingMedAbsErr **=** **NA**,

TestingMedAbsErr **=** **NA**,

Diff **=** **NA**,

TrainMeanAbsErr **=** **NA**,

TestMeanAbsErr **=** **NA**,

TrainMedianAbsErr **=** **NA**,

TestMedianAbsErr **=** **NA**,

TrainMedianRelativeErrPerc **=** **NA**,

TestMedianRelativeErrPerc **=** **NA**,

TrainMeanRelativeErrPerc **=** **NA**,

TestMeanRelativeErrPerc **=** **NA**,

RSquaredValueTest **=** **NA**,

RSquaredValueTrain **=** **NA**,

TTestSignificance **=** **NA)**

ModelResults**$**ChosenSeed **=** SeedNum

ModelResults**$**TrainingCor **=** cor.test**(**x **=** dataTRAINX**$**actual, y **=** dataTRAINX**$**pred**)$**estimate

ModelResults**$**TestingCor **=** cor.test**(**x **=** dataTESTX**$**actual, y **=** dataTESTX**$**pred**)$**estimate

ModelResults**$**TrainingMedAbsErr **=** median**(**abs**(**dataTRAINX**$**actual **-** dataTRAINX**$**pred**))**

ModelResults**$**TestingMedAbsErr **=** median**(**abs**(**dataTESTX**$**actual **-** dataTESTX**$**pred**))**

ModelResults**$**Diff **=** sum**(**ModelResults**$**TrainingMedAbs **-** ModelResults**$**TestingMedAbs**)**

ModelResults**$**TrainMeanAbsErr **=** Model_TrainMeanAE

ModelResults**$**TestMeanAbsErr **=** Model_TestMeanAE

ModelResults**$**TrainMedianAbsErr **=** Model_TrainMedAE

ModelResults**$**TestMedianAbsErr **=** Model_TestMedAE

ModelResults**$**TrainMedianRelativeErrPerc **=** median**(**Model_TrainRelErr**)**

ModelResults**$**TestMedianRelativeErrPerc **=** median**(**Model_TestRelErr**)**

ModelResults**$**TrainMeanRelativeErrPerc **=** mean**(**Model_TrainRelErr**)**

ModelResults**$**TestMeanRelativeErrPerc **=** mean**(**Model_TestRelErr**)**

ModelResults**$**RSquaredValueTest **=** as.numeric**(**R2Test**)**

ModelResults**$**RSquaredValueTrain **=** as.numeric**(**R2Train**)**

ModelResults**$**TTestSignificance **=** XTTestResult**$**p.value

### Writes model summary to a CSV file - Changes name to whichever summary file it is for

write.csv**(**ModelResults, paste0**(**wdinput, "ModelSummary", DatasetUsed, ".csv"**)**, row.names **=** **FALSE)**

### Comparison of Correlation Between species RelErr and Percentage non zeros of promoter blasts ###

# Make DF of Train RelErr Species

TrainRelErrPercZeros **<-** as.data.frame**(**datTrainPredict**)**

### Rename the s1 column to RelErr

TrainRelErrPercZeros **<-** TrainRelErrPercZeros %>% mutate**(**Predict **=** s1**)**

TrainRelErrPercZeros**$**s1 **<-** **NULL**

### Add in the extra info from the data frame without species names, but with the other parts

TrainRelErrPercZeros **<-** TrainRelErrPercZeros %>%

mutate**(**RelErr **=** dataTRAINX**$**RelErr**)**

TrainRelErrPercZeros **<-** TrainRelErrPercZeros %>%

mutate**(**Actual **=** dataTRAINX**$**actual**)**

### Iterate over row names in TrainRelErrPercNonZeros

**for** **(**row_name **in** rownames**(**TrainRelErrPercZeros**))** **{**

### Find matching row in dfPerNonZero based on Species column

matching_row **<-** dfPerZero**[**dfPerZero**$**Species **==** row_name, **]**

### Check if matching row exists

**if** **(**nrow**(**matching_row**)** **>** 0**)** **{**

### Get the corresponding value from Percentage column

percentage_value **<-** matching_row**$**Percentage

### Append the value to TrainRelErrPercNonZeros

TrainRelErrPercZeros**[**row_name, "PercentageZeros"**]** **<-** percentage_value

**}**

**}**

### Perform correlation test

corRelErrZeroTrain **<-** cor.test**(**TrainRelErrPercZeros**$**RelErr, TrainRelErrPercZeros**$**PercentageZeros**)**

### Calculate R-squared value

r_squaredRelErrZeroTrain **<-** corRelErrZeroTrain**$**estimate**^**2

### Create ggplot

ggplot**(**TrainRelErrPercZeros, aes**(**x **=** RelErr, y **=** PercentageZeros**))** **+**

geom_point**()** **+**

geom_text**(**x **=** max**(**TrainRelErrPercZeros**$**RelErr**)**, y **=** max**(**TrainRelErrPercZeros**$**PercentageZeros**)**,

label **=** paste**(**"Correlation:", round**(**corRelErrZeroTrain**$**estimate, 2**)**,

"\nR-squared:", round**(**r_squaredRelErrZeroTrain, 2**)**,

"\nSignificance:", ifelse**(**corRelErrZeroTrain**$**p.value **<** 0.001, "< 0.001", round**(**corRelErrZeroTrain**$**p.value, 3**)))**,

hjust **=** 1, vjust **=** 1**)**

### Make DF of Test RelErr Species

TestRelErrPercZeros **<-** as.data.frame**(**datTestPredict**)**

### Rename the s1 column to RelErr

TestRelErrPercZeros **<-** TestRelErrPercZeros %>% mutate**(**Predict **=** s1**)**

TestRelErrPercZeros**$**s1 **<-** **NULL**

### Add in the extra info from the data frame without species names, but with the other parts

TestRelErrPercZeros **<-** TestRelErrPercZeros %>%

mutate**(**RelErr **=** dataTESTX**$**RelErr**)**

TestRelErrPercZeros **<-** TestRelErrPercZeros %>%

mutate**(**Actual **=** dataTESTX**$**actual**)**

### Iterate over row names in TestRelErrPercNonZeros

**for** **(**row_name **in** rownames**(**TestRelErrPercZeros**))** **{**

### Find matching row in dfPerNonZero based on Species column

matching_row **<-** dfPerZero**[**dfPerZero**$**Species **==** row_name, **]**

### Check if matching row exists

**if** **(**nrow**(**matching_row**)** **>** 0**)** **{**

### Get the corresponding value from Percentage column

percentage_value **<-** matching_row**$**Percentage

### Append the value to TestRelErrPercNonZeros

TestRelErrPercZeros**[**row_name, "PercentageZeros"**]** **<-** percentage_value

**}**

**}**

### Perform correlation test

corRelErrZeroTest **<-** cor.test**(**TestRelErrPercZeros**$**RelErr, TestRelErrPercZeros**$**PercentageZeros**)**

### Calculate R-squared value

r_squaredRelErrZeroTest **<-** corRelErrZeroTest**$**estimate**^**2

### Create ggplot

ggplot**(**TestRelErrPercZeros, aes**(**x **=** RelErr, y **=** PercentageZeros**))** **+**

geom_point**()** **+**

geom_text**(**x **=** max**(**TestRelErrPercZeros**$**RelErr**)**, y **=** max**(**TestRelErrPercZeros**$**PercentageZeros**)**,

label **=** paste**(**"Correlation:", round**(**corRelErrZeroTest**$**estimate, 2**)**,

"\nR-squared:", round**(**r_squaredRelErrZeroTest, 2**)**,

"\nSignificance:", ifelse**(**corRelErrZeroTest**$**p.value **<** 0.001, "< 0.001", round**(**corRelErrZeroTest**$**p.value, 3**)))**,

hjust **=** 1, vjust **=** 1**)**

################################

### Gene Ontology Enrichment ###

################################

### Gene ontology work to get genes used in model ###

### Utilise Dat promoters file, which has identified the promoter sequences for the model

### Also need the HG19 Promoter Fasta file

### Make datPromoters non atomic

datPromotersX **<-** as.data.frame**(**datPROMOTERS**)**

### The below gets the iteration number for a later loop by counting the number of promoters used

NumProm **=** **(**nrow**(**datPromotersX**)-**1**)**

### Make a new dataframe for the promoters and gene names - different numbers of rows are needed based on the number of promoters

ModelPromoters **<-** data.frame**(**Number **=** 1**:(**NumProm**+**1**)**, PromoterID **=** **NA**, GeneName **=** **NA)**

### Make the promoter ID a column, not the rowname

datPromotersX **<-** tibble**::**rownames_to_column**(**datPromotersX, "PromoterID"**)**

### Add the Promoter Ids to the new dataframe

ModelPromoters**$**PromoterID **<-** datPromotersX**$**PromoterID

### Load in fasta file - Must save as a csv, with no .fa at the end - this is the search file

# Must also add a row called Name, so the first promoter can be searched

HG19 **<-** read.csv**(**paste0**(**wdinput, "hg19_ZLwnj.csv"**))**

### Make a dataframe of the HG19 promoter info

HG19X **<-** as.data.frame**(**HG19**)**

### Remove the intercept row, which must be done to allow the following commands

ModelPromoters **<-** ModelPromoters**[-**c**(**1**)**, **]**

### A loop to use the number of promoters to copy the correct gene identifiers and names

**for(**i **in** 1**:**NumProm**){**

### The strdetect command searches for the match to the ID, and appends that one

# Adds to the promoter file the text copied for each FP, which have the gene name present

ModelPromoters**[**i,3**]** **<-** HG19X**[**str_detect**(**HG19X**$**NAME, ModelPromoters**[**i,2**])**, **]** # Extract matching rows with str_detect

print**(**i**)** # Shows a running print of which i number it has reached

**}**

### Remove the unneeded info before genename, using the first space as the identifier

# ^indicates the first space, then all before first space is removed

ModelPromoters**$**GeneName **<-** sub**(**"^[^ ]* ", "", ModelPromoters**$**GeneName**)**

### Remove the unneeded info after genename, using the _1 as the identifier

# \\ignores all up to the_1, while .*indicates hit all including the _1

ModelPromoters**$**GeneName **<-** sub**(**"\\_1.*", "", ModelPromoters**$**GeneName**)**

### Now ModelPromoters can be used for Gene Ontology using the list in GeneNames

# Save as a CSV below to export list of genenames for ontology - Change the naming and path where required

write.csv**(**ModelPromoters, paste0**(**wdinput, "GeneNames", DatasetUsed, ".csv"**)**, row.names **=** **FALSE)**

### Downloads the Enrichr database

dbs **<-** listEnrichrDbs**()**

dbs **<-** c**(**"GO_Molecular_Function_2018", "GO_Cellular_Component_2018", "GO_Biological_Process_2018" , "ChEA_2016" ,"KEGG_2016"**)**

### Correlated significant genes

# Edit the below

GeneNames **<-** read.csv**(**paste0**(**wdinput, "GeneNames", DatasetUsed, ".csv"**))**

datGenes **<-** enrichr**(**genes **=** as.character**(**GeneNames**$**GeneName**)**, dbs**)**

### Export each GO function into a seperate table

datMolecular **<-** datGenes**$**GO_Molecular_Function_2018

datCellular **<-** datGenes**$**GO_Cellular_Component_2018

datBiological **<-** datGenes**$**GO_Biological_Process_2018

### Subset with significant results only

SigMolecular **<-** subset**(**datMolecular, datMolecular**$**Adjusted.P.value**<**0.05**)**

SigCellular **<-** subset**(**datCellular, datCellular**$**Adjusted.P.value**<**0.05**)**

SigBiological **<-** subset**(**datBiological, datBiological**$**Adjusted.P.value**<**0.05**)**

### Add a -log 10 adjusted p value column, where old p value column was

SigMolecular**[**5**]** **<-** **-**log10**(**SigMolecular**[**4**])**

SigCellular**[**5**]** **<-** **-**log10**(**SigCellular**[**4**])**

SigBiological**[**5**]** **<-** **-**log10**(**SigBiological**[**4**])**

colnames**(**SigMolecular**)[**5**]** **<-** "Adjlog10p"

colnames**(**SigCellular**)[**5**]** **<-** "Adjlog10p"

colnames**(**SigBiological**)[**5**]** **<-** "Adjlog10p"

colnames**(**SigMolecular**)[**1**]** **<-** "Function"

colnames**(**SigCellular**)[**1**]** **<-** "Function"

colnames**(**SigBiological**)[**1**]** **<-** "Function"

### Clean up function names

SigMolecular**$**Function **<-** sub**(**"\\(GO.*", "", SigMolecular**$**Function**)**

SigCellular**$**Function **<-** sub**(**"\\(GO.*", "", SigCellular**$**Function**)**

SigBiological**$**Function **<-** sub**(**"\\(GO.*", "", SigBiological**$**Function**)**

### Save as a CSV below to export list of GO results - Change the naming and path where required

write.csv**(**datMolecular, paste0**(**wdinput, "GOMolecular", DatasetUsed, ".csv"**)**, row.names **=** **FALSE)**

write.csv**(**datCellular, paste0**(**wdinput, "GOCellular", DatasetUsed, ".csv"**)**, row.names **=** **FALSE)**

write.csv**(**datBiological, paste0**(**wdinput, "GOBiological", DatasetUsed, ".csv"**)**, row.names **=** **FALSE)**

write.csv**(**SigMolecular, paste0**(**wdinput, "SigGOMolecular", DatasetUsed, ".csv"**)**, row.names **=** **FALSE)**

write.csv**(**SigCellular, paste0**(**wdinput, "SigGOCellular", DatasetUsed, ".csv"**)**, row.names **=** **FALSE)**

write.csv**(**SigBiological, paste0**(**wdinput, "SigGOBiological", DatasetUsed, ".csv"**)**, row.names **=** **FALSE)**

#########################

### PLOTS AND FIGURES ###

#########################

### Model Correlation Plots ###

### GGplot work

# More visually appealing graphs, using gg plot and the non atomic dataset, and viridis

### Plot the testing dataset

# Check if DatasetUsed contains the word "Ratio", and dynamically change labels

**if** **(**grepl**(**"Ratio", DatasetUsed**))** **{**

x_label **<-** "Actual ASM versus Lifespan Ratio"

y_label **<-** "Predicted ASM versus Lifespan Ratio"

y_labelBoxplot **<-** "Absolute Error (ASM versus Lifespan Ratio)"

**}** **else** **{**

x_label **<-** "Actual ASM (Days)"

y_label **<-** "Predicted ASM (Days)"

y_labelBoxplot **<-** "Absolute Error (Days)"

**}**

CorrelationGraphTest **<-** ggplot**(**dataTESTX, aes**(**x**=** actual, y**=** pred, fill**=** RelErr, label**=**pdTEST**$**Species**))+**

#geom_point(color='#E41A1C') +

geom_point**(**pch **=** 21, size **=** 3**)** **+** #Chooses fillable points

labs**(**x**=**x_label, y **=** y_label, title **=** "Testing Dataset"**)** **+**

geom_smooth**(**method**=**'lm',colour**=**'grey30',fill **=** "grey"**)** **+**

theme_bw**()** **+**

scale_fill_viridis_c**(**option **=** ViridisSetting, #Nice colour palette, picked by the option

direction **=** 1, #-1 swaps colour gradient

trans **=** "log",

breaks **=** c**(**1, 10, 100**)**,

name **=** "Relative\nerror"**)** **+**

theme**(**plot.title **=** element_text**(**hjust **=** 0.5**)**, panel.border **=** element_blank**()**,

panel.grid.major **=** element_blank**()**, panel.grid.minor **=** element_blank**()**,

axis.line **=** element_line**(**colour **=** "black"**))**

### Use the below to add names to the graph if wanted

# geom_text_repel(box.padding=1, size=1.5, max.overlaps = 10000)

print**(**CorrelationGraphTest**)**

# Plot output

ggsave**((**paste0**(**wdinput, "TestingPlot", DatasetUsed, ".pdf"**))**, CorrelationGraphTest, width **=** 8.27, height **=** 11.69**/**2, units **=** "in", dpi **=** 300**)**

saveRDS**(**CorrelationGraphTest, file **=** paste0**(**wdinput, "CorrelationGraphTest", DatasetUsed, ".rds"**))**

print**(**CorrelationGraphTest**)**

### Plot the training set - Remember, for ratio datasets, change the label for x and y to ratios, not days

CorrelationGraphTrain **<-** ggplot**(**dataTRAINX, aes**(**x**=** actual, y**=** pred, fill **=** RelErr, label**=**pdTRAIN**$**Species**))+**

geom_point**(**pch **=** 21, size **=** 3**)** **+** #Chooses fillable points

labs**(**x**=**x_label, y **=** y_label,title **=** "Training Dataset"**)** **+**

geom_smooth**(**method**=**'lm',colour**=**'grey30', fill**=**"grey"**)** **+**

theme_bw**()** **+**

scale_fill_viridis_c**(**option **=** ViridisSetting, #Nice colour palette, picked by the option

direction **=** 1, #-1 swaps colour gradient

trans **=** "log",

breaks **=** c**(**1, 10, 100**)**,

name **=** "Relative\nerror"**)** **+**

theme**(**plot.title **=** element_text**(**hjust **=** 0.5**)**, panel.border **=** element_blank**()**,

panel.grid.major **=** element_blank**()**, panel.grid.minor **=** element_blank**()**,

axis.line **=** element_line**(**colour **=** "black"**))**

### Plot output

ggsave**((**paste0**(**wdinput, "TrainingPlot", DatasetUsed, ".pdf"**))**, CorrelationGraphTrain, width **=** 8.27, height **=** 11.69**/**2, units **=** "in", dpi **=** 300**)**

saveRDS**(**CorrelationGraphTrain, file **=** paste0**(**wdinput, "CorrelationGraphTrain", DatasetUsed, ".rds"**))**

print**(**CorrelationGraphTrain**)**

### Absolute error boxplots ###

### Create new dataframes for Test and Train absolute error, needs to be separate dataframes#

AbsErrDataTrain **<-** data.frame**(**Data **=** dataTRAINX**[**, c**(**3**)]**, Type **=** 'Training Samples'**)**

AbsErrDataTest **<-** data.frame**(**Data **=** dataTESTX**[**, c**(**3**)]**, Type **=** 'Test Samples'**)**

### Combine frames to then plot, using a NEW df#

AbsErrDataPlot **<-** rbind**(**AbsErrDataTest, AbsErrDataTrain**)**

### Below is the boxplot code, outliers have been made "invisible" otherwise show as two points

# The guides line removes the legend

AbsErrBoxplot **<-** ggplot**(**AbsErrDataPlot, aes**(**x **=** Type, y **=** Data, colour **=** Type**))** **+**

stat_boxplot**(**geom **=** "errorbar", width **=** 0.2, color **=** "black"**)** **+**

geom_boxplot**(**outlier.shape **=** **NA**, color **=** "black", fill **=** "#F0F0F0"**)** **+** #Adds light grey background

geom_jitter**(**position **=** position_jitter**(**0.2**)**, shape **=** 16**)** **+**

labs**(**x **=** 'Sample Group', y **=** y_labelBoxplot, colour **=** "Sample Group"**)** **+**

scale_colour_brewer**(**palette **=** "Set1"**)** **+**

theme_bw**()** **+**

theme**(**panel.border **=** element_blank**()**,

panel.grid **=** element_blank**()**,

axis.line **=** element_line**(**colour **=** "black"**)**,

legend.position **=** "none"**)**

### Plot output, also as RDS file

ggsave**((**paste0**(**wdinput, "AbsErrPlot", DatasetUsed, ".pdf"**))**, AbsErrBoxplot, width **=** 8.27, height **=** 11.69**/**2, units **=** "in", dpi **=** 300**)**

saveRDS**(**AbsErrBoxplot, file **=** paste0**(**wdinput, "AbsErrPlot", DatasetUsed, ".rds"**))**

print**(**AbsErrBoxplot**)**

### GO Plots ###

### Make ggplots for all significant values, only if some are sig

# Use a -log10 of the adjusted p value and just show the significant results in a bar plot

# Use 1.3 as the cutoff, with a dotted line at 1.3 as this is 0.05 cutoof using -log10

**if** **(**nrow**(**SigBiological**)** **>**0**)**

**{**PlotBio **<-** ggplot**(**SigBiological, aes**(**x**=** Function, y**=** Adjlog10p**))** **+**

geom_bar**(**stat**=**"identity", width **=** 0.4**)** **+**

coord_flip**()** **+**

labs**(**title **=** "Biological Functional Significance"**)** **+**

xlab**(**"Function"**)** **+**

ylab**(**"Adjusted -log10 p value"**)** **+**

theme_bw**()** **+**

geom_hline**(**yintercept **=** **-**log10**(**0.05**)**, linetype **=** "dashed", color **=** "red", size **=** 0.75**)+**

scale_y_continuous**(**expand **=** c**(**0, 0**)**, limits **=** c**(**0, ceiling**((**max**(**SigBiological**[**5**])))))**

ggsave**((**paste0**(**wdinput, "GOBioPlot", DatasetUsed, ".pdf"**))**, PlotBio, width **=** 8.27, height **=** 11.69**/**2, units **=** "in", dpi **=** 300**)**

saveRDS**(**PlotBio, file **=** paste0**(**wdinput, "GOBioPlot", DatasetUsed, ".rds"**))**

print**(**PlotBio**)**

**}**

**if** **(**nrow**(**SigMolecular**)** **>**0**)**

**{**PlotMol **<-** ggplot**(**SigMolecular, aes**(**x**=** Function, y**=** Adjlog10p**))** **+**

geom_bar**(**stat**=**"identity", width **=** 0.4**)** **+**

coord_flip**()** **+**

labs**(**title **=** "Molecular Functional Significance"**)** **+**

xlab**(**"Function"**)** **+**

ylab**(**"Adjusted -log10 p value"**)** **+**

theme_bw**()** **+**

geom_hline**(**yintercept **=** **-**log10**(**0.05**)**, linetype **=** "dashed", color **=** "red", size **=** 0.75**)+**

scale_y_continuous**(**expand **=** c**(**0, 0**)**, limits **=** c**(**0, ceiling**((**max**(**SigMolecular**[**5**])))))**

ggsave**((**paste0**(**wdinput, "GOMolPlot", DatasetUsed, ".pdf"**))**, PlotMol, width **=** 8.27, height **=** 11.69**/**2, units **=** "in", dpi **=** 300**)**

saveRDS**(**PlotMol, file **=** paste0**(**wdinput, "GOMolPlot", DatasetUsed, ".rds"**))**

print**(**PlotMol**)**

**}**

**if** **(**nrow**(**SigCellular**)** **>**0**)**

**{**PlotCell **<-** ggplot**(**SigCellular, aes**(**x**=** Function, y**=** Adjlog10p**))** **+**

geom_bar**(**stat**=**"identity", width **=** 0.4**)** **+**

coord_flip**()** **+**

labs**(**title **=** "Cellular Functional Significance"**)** **+**

xlab**(**"Function"**)** **+**

ylab**(**"Adjusted -log10 p value"**)** **+**

theme_bw**()** **+**

geom_hline**(**yintercept **=** **-**log10**(**0.05**)**, linetype **=** "dashed", color **=** "red", size **=** 0.75**)+**

scale_y_continuous**(**expand **=** c**(**0, 0**)**, limits **=** c**(**0, ceiling**((**max**(**SigCellular**[**5**])))))**

ggsave**((**paste0**(**wdinput, "GOCellPlot", DatasetUsed, ".pdf"**))**, PlotCell, width **=** 8.27, height **=** 11.69**/**2, units **=** "in", dpi **=** 300**)**

saveRDS**(**PlotCell, file **=** paste0**(**wdinput, "GOCellPlot", DatasetUsed, ".rds"**))**

print**(**PlotCell**)**

**}**

### Plots the top 5 of all plots, and also gives and ABCDE labeling option

# Makes usable plots without text pushing off margins

**if** **(**nrow**(**SigBiological**)** **>** 0**)** **{**

top5_SigBiological **<-** head**(**arrange**(**SigBiological, desc**(**Adjlog10p**))**, 5**)**

# Reorder the Function variable based on the Adjlog10p values in descending order

top5_SigBiological**$**Function **<-** factor**(**top5_SigBiological**$**Function, levels **=** top5_SigBiological**$**Function**)**

PlotBio **<-** ggplot**(**top5_SigBiological, aes**(**x **=** Function, y **=** Adjlog10p**))** **+**

geom_bar**(**stat **=** "identity", width **=** 0.4**)** **+**

coord_flip**()** **+**

labs**(**title **=** "Biological Functional Significance"**)** **+**

xlab**(**"Function"**)** **+**

ylab**(**"Adjusted -log10 p value"**)** **+**

theme_bw**()** **+**

geom_hline**(**yintercept **=** **-**log10**(**0.05**)**, linetype **=** "dashed", color **=** "red", size **=** 0.75**)** **+**

scale_y_continuous**(**expand **=** c**(**0, 0**)**, limits **=** c**(**0, ceiling**(**max**(**top5_SigBiological**$**Adjlog10p**))))**

ggsave**((**paste0**(**wdinput, "GOBioPlotTop5", DatasetUsed, ".pdf"**))**, PlotBio, width **=** 8.27, height **=** 11.69**/**2, units **=** "in", dpi **=** 300**)**

saveRDS**(**PlotBio, file **=** paste0**(**wdinput, "GOBioPlotTop5", DatasetUsed, ".rds"**))**

print**(**PlotBio**)**

**}**

**if** **(**nrow**(**SigBiological**)** **>** 0**)** **{**

# Sort the data in descending order of Adjlog10p and take the top 5 rows

top5_SigBiological **<-** head**(**arrange**(**SigBiological, desc**(**Adjlog10p**))**, 5**)**

# Reorder the Function variable based on the Adjlog10p values in descending order

top5_SigBiological**$**Function **<-** factor**(**top5_SigBiological**$**Function, levels **=** top5_SigBiological**$**Function**)**

# Check if the dataframe has less than 5 rows

num_rows **<-** nrow**(**top5_SigBiological**)**

max_labels **<-** min**(**num_rows, 5**)** # Choose the minimum between 5 and the number of rows

# Create a vector with the labels from 'A' to 'E'

label_vector **<-** LETTERS**[**1**:**max_labels**]**

# Add a new column 'Label' to 'top5_SigBiological' with the label_vector

top5_SigBiological **<-** top5_SigBiological %>%

mutate**(**Label **=** label_vector**)**

PlotBio **<-** ggplot**(**top5_SigBiological, aes**(**x **=** Label, y **=** Adjlog10p**))** **+**

geom_bar**(**stat **=** "identity", width **=** 0.4**)** **+**

coord_flip**()** **+**

labs**(**title **=** "Biological Functional Significance"**)** **+**

xlab**(**"Top Five Functions"**)** **+**

ylab**(**"Adjusted -log10 p value"**)** **+**

theme_bw**()** **+**

geom_hline**(**yintercept **=** **-**log10**(**0.05**)**, linetype **=** "dashed", color **=** "red", size **=** 0.75**)** **+**

scale_y_continuous**(**expand **=** c**(**0, 0**)**, limits **=** c**(**0, ceiling**(**max**(**top5_SigBiological**$**Adjlog10p**)))**,

breaks **=** seq**(**0, ceiling**(**max**(**top5_SigBiological**$**Adjlog10p**))**, by **=** 0.5**))** **+**

scale_x_discrete**(**labels **=** label_vector**)** # Add scale for X-axis with the label_vector

ggsave**((**paste0**(**wdinput, "GOBioPlotTop5Letter", DatasetUsed, ".pdf"**))**, PlotBio, width **=** 8.27, height **=** 11.69**/**2, units **=** "in", dpi **=** 300**)**

saveRDS**(**PlotBio, file **=** paste0**(**wdinput, "GOBioPlotTop5Letters", DatasetUsed, ".rds"**))**

print**(**PlotBio**)**

**}**

**if** **(**nrow**(**SigMolecular**)** **>** 0**)** **{**

top5_SigMolecular **<-** head**(**arrange**(**SigMolecular, desc**(**Adjlog10p**))**, 5**)**

# Reorder the Function variable based on the Adjlog10p values in descending order

top5_SigMolecular**$**Function **<-** factor**(**top5_SigMolecular**$**Function, levels **=** top5_SigMolecular**$**Function**)**

PlotMol **<-** ggplot**(**top5_SigMolecular, aes**(**x **=** Function, y **=** Adjlog10p**))** **+**

geom_bar**(**stat **=** "identity", width **=** 0.4**)** **+**

coord_flip**()** **+**

labs**(**title **=** "Molecular Functional Significance"**)** **+**

xlab**(**"Function"**)** **+**

ylab**(**"Adjusted -log10 p value"**)** **+**

theme_bw**()** **+**

geom_hline**(**yintercept **=** **-**log10**(**0.05**)**, linetype **=** "dashed", color **=** "red", size **=** 0.75**)** **+**

scale_y_continuous**(**expand **=** c**(**0, 0**)**, limits **=** c**(**0, ceiling**(**max**(**top5_SigMolecular**$**Adjlog10p**))))**

ggsave**((**paste0**(**wdinput, "GOMolPlotTop5", DatasetUsed, ".pdf"**))**, PlotMol, width **=** 8.27, height **=** 11.69**/**2, units **=** "in", dpi **=** 300**)**

saveRDS**(**PlotMol, file **=** paste0**(**wdinput, "GOMolPlotTop5", DatasetUsed, ".rds"**))**

print**(**PlotMol**)**

**}**

**if** **(**nrow**(**SigMolecular**)** **>** 0**)** **{**

# Sort the data in descending order of Adjlog10p and take the top 5 rows

top5_SigMolecular **<-** head**(**arrange**(**SigMolecular, desc**(**Adjlog10p**))**, 5**)**

# Reorder the Function variable based on the Adjlog10p values in descending order

top5_SigMolecular**$**Function **<-** factor**(**top5_SigMolecular**$**Function, levels **=** top5_SigMolecular**$**Function**)**

# Check if the dataframe has less than 5 rows

num_rows **<-** nrow**(**top5_SigMolecular**)**

max_labels **<-** min**(**num_rows, 5**)** # Choose the minimum between 5 and the number of rows

# Create a vector with the labels from 'A' to 'E'

label_vector **<-** LETTERS**[**1**:**max_labels**]**

# Add a new column 'Label' to 'top5_SigMolecular' with the label_vector

top5_SigMolecular **<-** top5_SigMolecular %>%

mutate**(**Label **=** label_vector**)**

PlotMol **<-** ggplot**(**top5_SigMolecular, aes**(**x **=** Label, y **=** Adjlog10p**))** **+**

geom_bar**(**stat **=** "identity", width **=** 0.4**)** **+**

coord_flip**()** **+**

labs**(**title **=** "Molecular Functional Significance"**)** **+**

xlab**(**"Top Five Functions"**)** **+**

ylab**(**"Adjusted -log10 p value"**)** **+**

theme_bw**()** **+**

geom_hline**(**yintercept **=** **-**log10**(**0.05**)**, linetype **=** "dashed", color **=** "red", size **=** 0.75**)** **+**

scale_y_continuous**(**expand **=** c**(**0, 0**)**, limits **=** c**(**0, ceiling**(**max**(**top5_SigMolecular**$**Adjlog10p**)))**,

breaks **=** seq**(**0, ceiling**(**max**(**top5_SigMolecular**$**Adjlog10p**))**, by **=** 0.5**))** **+**

scale_x_discrete**(**labels **=** label_vector**)** # Add scale for X-axis with the label_vector

ggsave**((**paste0**(**wdinput, "GOMolPlotTop5Letters", DatasetUsed, ".pdf"**))**, PlotMol, width **=** 8.27, height **=** 11.69**/**2, units **=** "in", dpi **=** 300**)**

saveRDS**(**PlotMol, file **=** paste0**(**wdinput, "GOMolPlotTop5Letters", DatasetUsed, ".rds"**))**

print**(**PlotMol**)**

**}**

**if** **(**nrow**(**SigCellular**)** **>** 0**)** **{**

top5_SigCellular **<-** head**(**arrange**(**SigCellular, desc**(**Adjlog10p**))**, 5**)**

# Reorder the Function variable based on the Adjlog10p values in descending order

top5_SigCellular**$**Function **<-** factor**(**top5_SigCellular**$**Function, levels **=** top5_SigCellular**$**Function**)**

PlotCell **<-** ggplot**(**top5_SigCellular, aes**(**x **=** Function, y **=** Adjlog10p**))** **+**

geom_bar**(**stat **=** "identity", width **=** 0.4**)** **+**

coord_flip**()** **+**

labs**(**title **=** "Cellular Functional Significance"**)** **+**

xlab**(**"Function"**)** **+**

ylab**(**"Adjusted -log10 p value"**)** **+**

theme_bw**()** **+**

geom_hline**(**yintercept **=** **-**log10**(**0.05**)**, linetype **=** "dashed", color **=** "red", size **=** 0.75**)** **+**

scale_y_continuous**(**expand **=** c**(**0, 0**)**, limits **=** c**(**0, ceiling**(**max**(**top5_SigCellular**$**Adjlog10p**))))**

ggsave**((**paste0**(**wdinput, "GOCellPlotTop5", DatasetUsed, ".pdf"**))**, PlotCell, width **=** 8.27, height **=** 11.69**/**2, units **=** "in", dpi **=** 300**)**

saveRDS**(**PlotCell, file **=** paste0**(**wdinput, "GOCellPlotTop5", DatasetUsed, ".rds"**))**

print**(**PlotCell**)**

**}**

**if** **(**nrow**(**SigCellular**)** **>** 0**)** **{**

top5_SigCellular **<-** head**(**arrange**(**SigCellular, desc**(**Adjlog10p**))**, 5**)**

# Reorder the Function variable based on the Adjlog10p values in descending order

top5_SigCellular**$**Function **<-** factor**(**top5_SigCellular**$**Function, levels **=** top5_SigCellular**$**Function**)**

# Check if the dataframe has less than 5 rows

num_rows **<-** nrow**(**top5_SigCellular**)**

max_labels **<-** min**(**num_rows, 5**)** # Choose the minimum between 5 and the number of rows

# Create a vector with the labels from 'A' to 'E'

label_vector **<-** LETTERS**[**1**:**max_labels**]**

# Add a new column 'Label' to 'top5_SigCellular' with the label_vector

top5_SigCellular **<-** top5_SigCellular %>%

mutate**(**Label **=** label_vector**)**

PlotCell **<-** ggplot**(**top5_SigCellular, aes**(**x **=** Label, y **=** Adjlog10p**))** **+**

geom_bar**(**stat **=** "identity", width **=** 0.4**)** **+**

coord_flip**()** **+**

labs**(**title **=** "Cellular Functional Significance"**)** **+**

xlab**(**"Top Five Functions"**)** **+**

ylab**(**"Adjusted -log10 p value"**)** **+**

theme_bw**()** **+**

geom_hline**(**yintercept **=** **-**log10**(**0.05**)**, linetype **=** "dashed", color **=** "red", size **=** 0.75**)** **+**

scale_y_continuous**(**expand **=** c**(**0, 0**)**, limits **=** c**(**0, ceiling**(**max**(**top5_SigMolecular**$**Adjlog10p**)))**,

breaks **=** seq**(**0, ceiling**(**max**(**top5_SigMolecular**$**Adjlog10p**))**, by **=** 0.5**))** **+**

scale_x_discrete**(**labels **=** label_vector**)** # Add scale for X-axis with the label_vector

ggsave**((**paste0**(**wdinput, "GOCellPlotTop5Letters", DatasetUsed, ".pdf"**))**, PlotCell, width **=** 8.27, height **=** 11.69**/**2, units **=** "in", dpi **=** 300**)**

saveRDS**(**PlotCell, file **=** paste0**(**wdinput, "GOCellPlotTop5Letters", DatasetUsed, ".rds"**))**

print**(**PlotCell**)**

**}**

############################

### Relative Error Plots ###

############################

### Will only work if relative abs err exponentially decreasing with age

# Plot the relative error rates, gives clear indication of higher error with younger animals

# Only on the testing dataset

### Define the exponential function exp_funRelErr, used to model actual values and the RelErrvalues.

exp_funRelErr **<-** **function(**x, a, b**)** **{**

return**(**a ***** exp**(-**b ***** x**))**

**}**

### Fit the exponential function to the data

fitRelErr **<-** nls**(**RelErr **~** exp_funRelErr**(**actual, a, b**)**, data **=** dataTESTX, start **=** list**(**a **=** max**(**dataTESTX**$**RelErr**)**, b **=** 0.01**))**

### Define the equation label with the estimated parameter values

EquationLabel **<-** paste**(**"y = ", format**(**coef**(**fitRelErr**)[**1**]**, digits **=** 3**)**, " * exp(",

format**(**coef**(**fitRelErr**)[**2**]**, digits **=** 3**)**, " * x)"**)**

### Calculate the correlation coefficient between predicted and actual values

CorrelationRelErr **<-** cor**(**predict**(**fitRelErr**)**, dataTESTX**$**RelErr**)**

### Calculate the R-squared value of the fit

R2RelErr **<-** 1 **-** sum**(**residuals**(**fitRelErr**)^**2**)** **/** sum**((**dataTESTX**$**RelErr **-** mean**(**dataTESTX**$**RelErr**))^**2**)**

# Check if DatasetUsed contains the word "Ratio"

**if** **(**grepl**(**"Ratio", DatasetUsed**))** **{**

x_label **<-** "Ratio of ASM versus Lifespan"

**}** **else** **{**

x_label **<-** "Actual ASM Values in Days"

**}**

# Create the plot

PlotRelErr **<-** ggplot**(**dataTESTX, aes**(**x **=** actual, y **=** RelErr**))** **+**

geom_point**()** **+**

geom_smooth**(**method **=** "nls", formula **=** y **~** exp_funRelErr**(**x, a, b**)**, se **=** **FALSE**, color **=** "red",

method.args **=** list**(**start **=** coef**(**fitRelErr**)))** **+**

labs**(**x **=** x_label, y **=** "Relative Error as Percentage"**)** **+**

annotate**(**"text", x **=** max**(**dataTESTX**$**actual**)** ***** 0.6, y **=** max**(**dataTESTX**$**RelErr**)** ***** 0.8,

label **=** paste0**(**"Correlation: ", round**(**CorrelationRelErr, 3**)**, "\nR2: ", round**(**R2RelErr, 3**)))** **+**

geom_text**(**x **=** 1500, y **=** 210, label **=** as.character**(**EquationLabel**)**, parse **=** **FALSE)** **+**

theme_bw**()** **+** # Set the background to white

theme**(**panel.grid **=** element_blank**())** **+** # Remove panel grid lines (cross-hatching)

scale_x_continuous**()** **+** # Add x-axis scale

scale_y_continuous**()** # Add y-axis scale

# Save the plot as a PDF

ggsave**((**paste0**(**wdinput, "RelativeErrPlot", DatasetUsed, ".pdf"**))**, PlotRelErr, width **=** 8.27, height **=** 11.69**/**2, units **=** "in", dpi **=** 300**)**

saveRDS**(**PlotRelErr, file **=** paste0**(**wdinput, "RelativeErrPlot", DatasetUsed, ".rds"**))**

print**(**PlotRelErr**)**

#######################

### Script Complete ###

#######################
