# Supplementary figures and images for: A genomic predictor for age at sexual maturity for mammalian species
Source: Evol Appl. 2024 Jan 10;17(2):e13635. doi: 10.1111/eva.13635 (PMC10853647; doi:10.1111/eva.13635)

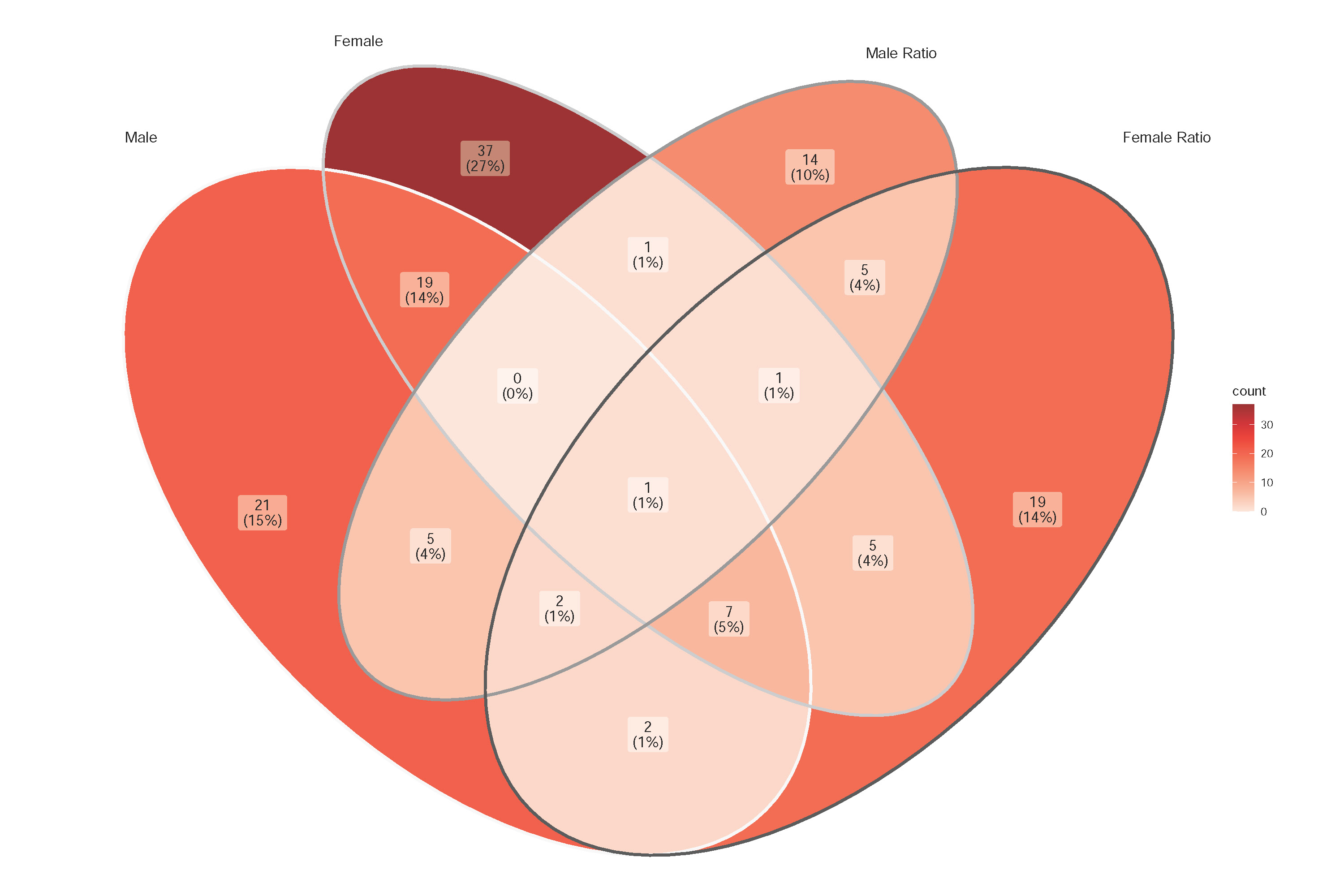

Supplement: Supplementary file 7 — Appendix S7. [file EVA-17-e13635-s001.jpg]
