# Supplementary material for: A genomic predictor for age at sexual maturity for mammalian species
Source: Evol Appl. 2024 Jan 10;17(2):e13635. doi: 10.1111/eva.13635 (PMC10853647; doi:10.1111/eva.13635)
Supplement: Supplementary file 13 — Data S1. [file EVA-17-e13635-s006.docx]

Supp 1 - The identity of species assessed in each of the four models, with the age of sexual maturity, lifespan, and ratio values used in the model recorded where applicable. All information was sourced from the AnAge database.

Supp 2 - R script for the four elastic net regression models which used CpG density in promoter regions to estimate sexual maturity in mammalian species.

Supp 3 - The percentage of non-zero CpG density BLAST hits against the human promoter set for species utilised in the male and female models.

Supp 4 - Model performance and assessment metrics of the four models.

Supp 5 - Gene names of the gene promoters utilised in the four models.

Supp 6 - Significant GO terms, with associated function/component/process and p values, for the GO analysis of the four models.

Supp 7 - Venn diagram of the shared number of gene promoters utilised by the four assessed models.

Supp 8 - Sequences of gene promoter regions of Homo sapiens, sourced from the Eukaryotic Promoter Database.

Supp 9 - Species names and sexual maturity values utilised by the female model. Sexual maturity age was sourced from the AnAge database.

Supp 10 - Species names and sexual maturity to lifespan ratio values utilised by the female ratio model. Sexual maturity age and lifespan was sourced from the AnAge database.

Supp 11 - Species names and sexual maturity values utilised by the male model. Sexual maturity age was sourced from the AnAge database.

Supp 12 - Species names and sexual maturity to lifespan ratio values utilised by the male ratio model. Sexual maturity age and lifespan was sourced from the AnAge database.
